# Influenza Vaccination Coverage and Predictors of Vaccination Intent Among Patients Undergoing Hemodialysis in Klang Valley, Malaysia: A Cross-Sectional Study

**DOI:** 10.7759/cureus.88564

**Published:** 2025-07-23

**Authors:** Voon Son Wong, Yu Hao Teh, Pooi Pooi Leong, Li Lian Tay, Norazinizah Ahmad Miswan, Teck Han Ng, Waye Hann Kang

**Affiliations:** 1 Department of Medicine, M. Kandiah Faculty of Medicine and Health Sciences, Universiti Tunku Abdul Rahman, Kajang, MYS; 2 Department of Preclinical Science, M. Kandiah Faculty of Medicine and Health Sciences, Universiti Tunku Abdul Rahman, Kajang, MYS; 3 Department of Nephrology, Hospital Sultan Idris Shah Serdang, Kementerian Kesihatan Malaysia, Serdang, MYS; 4 Department of Medicine, Ampang Hospital, Kementerian Kesihatan Malaysia, Ampang, MYS

**Keywords:** dialysis, influenza, influenza vaccine, knowledge, vaccination rate

## Abstract

Introduction

Influenza poses a significant risk to patients receiving hemodialysis, while vaccination reduces the risk of hospitalization, cardiovascular events, and mortality. However, the influenza vaccine uptake rate is low in low- and middle-income countries. This study aimed to assess influenza vaccination coverage, knowledge, and intentions regarding influenza vaccination, as well as to identify sociodemographic and clinical factors associated with vaccination intention among patients undergoing hemodialysis in the Klang Valley, Malaysia.

Materials and methods

An observational cross-sectional study was conducted in three private and two public dialysis centers in Malaysia between August and December 2024. Data were collected from patients receiving hemodialysis using a structured questionnaire. There were twelve questions with dichotomous scoring (correct = 1, incorrect/unsure = 0). The scores were later categorized as poor (0-4), moderate (5-7), and good (8-12), with the cut-off points based on tertile distribution. Statistical analyses were performed to determine associations between variables related to knowledge of influenza and its vaccine, as well as predictors of influenza vaccination intention.

Results

Only 10.8% of 298 respondents had been vaccinated at least once in their lifetime, despite 90% being aware of influenza and its vaccine. Social media (31.5%) and television (23.5%) were the two primary sources of information. The influenza vaccination intention rate is only 40.3%, despite 74.2% of respondents having moderate to good levels of knowledge about the vaccine. Good knowledge level (aOR=6.58, 95% CI 3.145-13.889, p<0.001), female gender (aOR=1.721, 95% CI 1.108-2.915, p=0.043), Chinese ethnicity (aOR=2.443, 95% CI 1.273-4.689, p=0.007), and recommendation by healthcare providers (aOR=3.64, 95% CI 1.497-8.836, p=0.004) are significant predictors for influenza vaccination intention.

Conclusions

This study highlights the significant misconceptions about influenza and its vaccine among patients receiving hemodialysis in Malaysia. Targeted public health campaigns and healthcare professional engagements are crucial in improving vaccination intention rates in this vulnerable population. Addressing these knowledge gaps could reduce influenza-related complications and mortality among these patients.

## Introduction

Influenza is a widespread respiratory infection that occurs globally and poses a significant risk to patients with end-stage renal failure (ESRF) as they have an impaired immune system and are at high risk of serious complications from influenza infection [1-4]. These patients have increased hospitalization and death rates, with up to 10-fold higher mortality rates compared to the general population.

Influenza vaccines reduce the risk of hospitalization in patients with chronic kidney disease [1,2]. Aside from that, the influenza vaccine has been shown to lower other inpatient complications, such as ICU admissions and venous thromboembolism events, and reduce the risk of major adverse cardiovascular events, all-cause mortality, cardiovascular mortality, and myocardial infarction in both patients receiving hemodialysis and peritoneal dialysis [3-9]. Timely vaccination and additional preventive measures are vital for ESRF patients, as they may have a lower immune response to the influenza vaccine [7,10].

Globally, influenza vaccination rates vary widely depending on the country, healthcare infrastructure, and public health initiatives. In many low- and middle-income countries, influenza vaccination rates are significantly lower, often ranging from 10% to 20% of the general population [11]. Vaccination rates tend to be lower in certain high-risk groups due to various factors, including lack of awareness, misconceptions about the vaccine’s safety, and concerns about vaccine side effects.

In Malaysia, there is currently no data available on influenza vaccination rates among patients undergoing hemodialysis. However, a nationwide study assessed influenza vaccination coverage among the elderly and patients with diabetes, finding rates of 5.5% and 6.4%, respectively [12]. As Malaysia initiates its free influenza vaccination program for high-risk citizens, it is crucial to assess the knowledge and awareness of these patients to enhance vaccination intention rates [13].

While several studies have examined healthcare workers’ knowledge of influenza and their attitudes toward its vaccine, research focusing on the high-risk patient populations, such as those undergoing dialysis, remains very limited. This study primarily aimed to (1) determine influenza vaccination coverage among patients receiving hemodialysis in Klang Valley and (2) assess knowledge levels regarding influenza and vaccination. Secondary objectives of the study include identifying (3) sociodemographic and clinical predictors of vaccination intention and (4) examining barriers to vaccination uptake.

## Materials and methods

This observational cross-sectional study was conducted between August and December 2024 across five dialysis centers in Selangor, Malaysia: three private facilities and two public facilities. These centers were selected to represent both public and private healthcare sectors in the Klang Valley.

We included participants who fulfilled these inclusion criteria: (1) ESRF patients aged ≥18 years, (2) receiving maintenance hemodialysis for ≥3 months, (3) able to provide informed consent, and (4) able to communicate in Bahasa Malaysia or English. They were excluded if they had (1) an acute illness requiring hospitalization, (2) cognitive impairment preventing informed consent, (3) dialysis duration of <3 months, and (4) refusal to participate.

Participants were recruited using convenience sampling. In each dialysis center, we approached consecutive eligible patients during their regular dialysis sessions over the study period. To minimize selection bias, recruitment took place across different days of the week and time slots. All eligible patients who presented during the data collection periods were invited to participate, and any reasons for non-participation were recorded. Based on a previous Malaysian study reporting 11.9% influenza vaccination uptake, we calculated the minimum required sample size using the formula for single proportion: $n = \frac{Z^2 \cdot p (1 - p)}{d^2}$, where Z = 1.96 (95% confidence level), p=0.119, and d=0.04 (margin of error) [12]. The calculated minimal sample size was 252 participants (95% CI, 4% margin of error). We recruited 298 participants (119% of the minimum required) to account for potential incomplete responses and improve study power. A total of 306 participants were approached, but eight were excluded due to language barriers (3), refusal to participate (3), and time constraints (2).

Questionnaire and data collection

Data collection was conducted by the authors and five trained research assistants during the participants' routine hemodialysis sessions. This was preceded by a four-hour training session on the study protocol and questionnaire administration, as well as practice sessions with standardized scenarios. An inter-rater reliability assessment achieved greater than 90% agreement. The questionnaire was administered (interviewer-led) in Malay and English, and questions were clarified without influencing responses. The average completion time ranged from 15 to 20 minutes, and all questionnaires were reviewed for completeness before participants departed. A daily review of completed questionnaires was conducted to ensure completeness.

The questionnaire comprised closed-response questions divided into four sections: (i) sociodemographic data, including age, gender, ethnicity, religion, educational level, marital status, and monthly income; (ii) information about influenza, including source of information or history of influenza vaccination; (iii) clinical parameters, including causes of ESRF, duration of dialysis, and presence of comorbidities; and (iv) respondents' knowledge of influenza and its vaccine. The knowledge questions were adapted from validated instruments, and content validity was assessed by three healthcare professionals: a nephrologist, a general physician, and a family physician [14-18]. Forward and back translation from English to Malay was performed by two independent translators, with discrepancies reconciled by a content expert. Cognitive interviews were conducted with 10 patients receiving hemodialysis to assess question comprehension, and modifications were subsequently made based on their feedback.

A pilot study was subsequently conducted among 40 healthy individuals, yielding an acceptable internal consistency (Cronbach's alpha = 0.916) for the knowledge questions. There were 12 questions with dichotomous scoring (correct = 1, incorrect/unsure = 0). The scores were later categorized into poor (0-4), moderate (5-7), and good (8-12), with the cut-off points based on the tertile distribution. The first cut-off score is the upper limit of the first tertile (33.33rd percentile), while the second cut-off score is the upper limit of the second tertile (the 66.66th percentile). The reason for choosing this cut-off is its ability to reflect natural groupings in the data, representing low, medium, and high levels of the measured construct, thereby providing a straightforward way to interpret scores.

The questionnaires were reviewed daily to ensure completeness, with 10% of the data randomly double-entered for verification purposes. No missing data points were detected during this study. The data collected were analyzed using SPSS Statistics version 24.0 (IBM Corp. Released 2016. IBM SPSS Statistics for Windows, Version 24.0. Armonk, NY: IBM Corp.). Statistical significance was set at p<0.05. Categorical variables were shown using frequencies and percentages. A chi-square test was performed to determine bivariate associations for categorical variables.

Statistical analysis

Descriptive statistics were used to show the distribution of patients' sociodemographic characteristics. Statistical analysis was performed on all demographic parameters for comparison, with p<0.05 considered statistically significant. Crosstabulation with the chi-square test of proportion was applied to assess the association between the respondents' sociodemographic characteristics and their knowledge of influenza and influenza vaccine.

Bivariate and logistic regression models were used to evaluate the odds ratio and predicting factors for vaccination intention (dependent variable). Variables with p<0.20 in univariate analysis were included in the initial model. The backward elimination method was used to remove variables with p>0.10. Statistical assumptions were checked, ensuring (i) linearity of continuous variables by checking the residual plots; (ii) independence of multicollinearity by ensuring VIF <5, tolerance >0.2; (iii) identification of outliers using standardized residuals; and (iv) model fit with the Hosmer-Lemeshow goodness-of-fit test.

Ethical approval and informed consent

This study was approved by the Universiti Tunku Abdul Rahman Ethics Committee (U/SERC/56(A)-413/2024) and the Medical Research and Ethics Committee (ID-24-03123-ART).

Written informed consent was obtained from all participants before data collection. Participants were informed that participation was voluntary and that they could withdraw at any time without affecting their medical care. All data were anonymized and stored securely following institutional data protection policies.

## Results

Of the 306 eligible participants (representing the total population of all five dialysis centers), 298 (97.4%) completed the study. Participants' mean age was 55.4±13.4 years, with 176 (59.1%) aged <60 years. Most participants were Malay (n=199, 66.8%), had a secondary education (n=174, 58.4%), and were unemployed (n=113, 37.9%). Most of the patients admitted that hypertension and/or diabetes were the main reasons for them ending up with dialysis; 16.1% (48/298) had diabetes, 29.9% (89/298) had hypertension, while 24.8% (74/298) had both hypertension and diabetes. A significant association was found between knowledge level and age groups (p<0.001), education level (p<0.001), and occupation (p=0.035) (Table 1). Vaccination rates were consistently low across all centers, with annual vaccination rates ranging from 0% to 4.9% (p=0.776, chi-square test).

**Table 1:** Sociodemographic and clinical parameters of the respondents according to knowledge level and intention to vaccinate (n=298) * Statistically significant at p<0.05

Variables		Total	Knowledge			p-value	Intention to be vaccinated		p-value
			Poor	Moderate	Good		Yes	No	
		(n=298)	(n=77)	(n=133)	(n=88)		(n=120)	(n=178)	
Age, year						<0.001*			0.11
<20		1 (0.3%)	0 (0.0%)	1 (0.6%)	0 (0.0%)		0 (0.0%)	1 (0.06%)	
20-29		9 (3.0%)	0 (0.0%)	2 (1.5%)	7 (8.0%)		7 (5.8%)	2 (0.11%)	
30-39		28 (9.4%)	4 (5.2%)	13 (9.8%)	11 (12.5%)		15 (12.5%)	13 (7.3%)	
40-49	61 (20.5%)		9 (11.7%)	31 (23.3%)	21 (23.9%)		27 (22.5%)	34 (19.1%)	
50-59	77 (25.8%)		23 (29.9%)	29 (21.8%)	25 (28.4%)		27 (22.5%)	50 (28%)	
60-69	80 (26.9%)		23 (29.9%)	44 (33.1%)	13 (14.8%)		30 (25.0%)	50 (28%)	
≥70	42 (14.1%)		18 (23.4%)	13 (9.8%)	11 (12.5%)		14 (11.7%)	28 (15.7%)	
Gender						0.248			0.131
Male	135 (45.3%)		30 (39.0%)	67 (50.4%)	38 (43.2%)		48 (40.0%)	87 (48.9%)	
Female	163 (54.7%)		47 (61.0%)	66 (49.6%)	50 (56.8%)		72 (60.0%)	91 (51.1%)	
Ethnicity						0.894			0.011*
Malay	199 (66.8%)		51 (66.2%)	89 (66.9%)	59 (67.0%)		91 (75.8%)	108 (60.7%)	
Chinese	66 (22.1%)		16 (20.8%)	29 (21.8%)	21 (23.9%)		19 (15.8%)	47 (26.4%)	
Indian	29 (9.7%)		9 (11.7%)	14 (10.5%)	6 (6.8%)		7 (5.8%)	22 (12.4%)	
Others	4 (1.3%)		1 (1.3%)	1 (0.8%)	2 (2.3%)		3 (2.5%)	1 (0.6%)	
Religion						0.747			0.57
Islam	201 (67.4%)		53 (68.8%)	89 (66.9%)	59 (67.1%)		90 (75.0%)	111 (62.4%)	
Buddhist	51 (17.1%)		13 (16.9%)	21 (15.8%)	17 (19.3%)		14 (11.7%)	37 (20.8%)	
Hindu	30 (10.1%)		9 (11.7%)	16 (12.0%)	5 (5.7%)		8 (6.7%)	22 (12.4%)	
Christian	8 (2.7%)		0 (0.0%)	4 (3.0%)	4 (4.5%)		5 (4.2%)	3 (1.7%)	
Other	8 (2.7%)		2 (2.6%)	3 (2.3%)	3 (3.4%)		3 (2.5%)	5 (2.8%)	
Education						<0.001*			<0.003*
No formal education	12 (4.0%)		8 (10.4%)	3 (2.3%)	1 (1.1%)		1 (0.8%)	11 (6.2%)	
Primary	43 (14.4%)		18 (23.4%)	18 (13.5%)	7 (8.0%)		15 (12.5%)	28 (15.7%)	
Secondary	174 (58.4%)		45 (58.4%)	79 (59.4%)	50 (56.8%)		65 (54.2%)	109 (61.2%)	
Tertiary	69 (23.2%)		6 (7.8%)	33 (24.8%)	30 (34.1%)		39 (32.5%)	30 (16.9%)	
Occupation						0.035			0.263
Unemployed	113 (37.9%)		34 (44.2%)	45 (33.8%)	34 (37.9%)		40 (33.3%)	73 (41.0%)	
Self-employed	27 (9.1%)		4 (5.2%)	10 (7.5%)	13 (14.8%)		15 (12.5%)	12 (6.7%)	
Employee	37 (12.4%)		4 (5.2%)	18 (13.5%)	15 (17.0%)		18 (15.0%)	19 (10.7%)	
Retired	117 (39.3%)		34 (44.2%)	59 (44.4%)	24 (27.3%)		46 (38.3%)	71 (39.9%)	
Student	4 (1.3%)		1 (1.3%)	1 (1.3%)	2 (2.3%)		1 (0.8%)	3 (1.7%)	
Income						0.086*			0.01*
B40	226 (75.8%)		64 (83.1%)	101 (75.9%)	61 (69.3%)		81 (67.5%)	145 (81.5%)	
M40	64 (21.5%)		12 (15.6%)	26 (19.5%)	26 (29.5%)		33 (27.5%)	31 (17.4%)	
T20	8 (2.7%)		1 (1.3%)	6 (4.5%)	1 (1.1%)		6 (5.0%)	2 (1.1%)	
Marital						0.383			0.734
Single	52 (17.4%)		8 (10.4%)	19 (14.3%)	25 (28.4%)		23 (19.2%)	29 (16.3%)	
Married	210 (70.5%)		56 (72.7%)	100 (75.2%)	54 (61.4%)		85 (70.8%)	125 (70.2%)	
Divorced	14 (4.7%)		4 (5.2%)	8 (6.0%)	2 (2.3%)		4 (3.3%)	10 (5.6%)	
Widowed	22 (7.4%)		9 (11.7%)	6 (4.5%)	7 (8.0%)		8 (6.7%)	14 (7.9%)	
Center						0.052			0.837
Private	171 (57.4%)		51 (66.2%)	78 (58.6%)	42 (47.7%)		68 (56.7%)	103 (57.9%)	
Government	127 (42.6%)		26 (33.8%)	55 (41.4%)	46 (52.5%)		52 (43.3%)	75 (42.1%)	
Causes of renal disease						0.016			0.077
Diabetes and hypertension	74 (24.8%)		18 (23.4%)	37 (27.8%)	19 (21.6%)		20 (16.7%)	54 (30.3%)	
Diabetes	48 (16.1%)		17 (22.1%)	20 (15.0%)	10 (11.4%)		19 (15.8%)	29 (16.3%)	
Hypertension	89 (29.9%)		26 (33.8%)	42 (31.6%)	22 (25.0%)		44 (36.7%)	45 (25.3%)	
Glomerular diseases	29 (9.7%)		2 (2.6%)	15 (11.3%)	12 (13.6%)		10 (8.3%)	19 (10.7%)	
Unknown	20 (6.7%)		7 (9.1%)	8 (6.0%)	5 (5.7%)		8 (6.7%)	12 (6.7%)	
Others	38 (12.8%)		7 (9.1%)	11 (8.3%)	20 (22.7%)		19 (15.8%)	19 (10.7%)	

Despite 90% of respondents being aware of influenza, only 10.8% had received the influenza vaccine, and 3.4% received the vaccination annually. They mainly received information on influenza and its vaccination through social media (31.5%), followed by television (23.5%) and other internet sources (7.7%). Only 10% of them had ever been recommended by their health care providers for influenza vaccinations. There is a significant association between knowledge level and awareness of influenza (p<0.001), a history of influenza vaccination (p<0.001), and sources of information on influenza and its vaccine (p=0.002) (Table 2).

**Table 2:** Basic information on influenza and its vaccine according to knowledge level and intention to vaccinate (n=298) * Statistically significant at p<0.05

Variables	Total	Knowledge			p-value	Intention to be vaccinated		p-value
		Poor	Moderate	Good		Yes	No	
	(n=298)	(n=77)	(n=133)	(n=88)		(n=120)	(n=178)	
Have you ever heard of influenza					<0.001*			0.005*
Yes	258 (86.6%)	56 (72.7%)	118 (88.7%)	84 (95.5%)		112 (93.3%)	146 (82.0%)	
No	40 (13.4%)	21 (27.3%)	15 (11.3%)	4 (4.5%)		8 (6.7%)	32 (18.0%)	
Have you been vaccinated for influenza annually					<0.001*			0.002*
Yes	10 (3.4%)	0 (0.0%)	5 (2.8%)	5 (11.4%)		6 (5.0%)	4 (2.2%)	
Yes, but not annually	22 (7.4%)	0 (0.0%)	9 (6.8%)	13 (14.3%)		16 (13.3%)	6 (3.4%)	
Never	266 (89.3%)	77 (100.0%)	119 (89.5%)	70 (79.5%)		98 (81.7%)	168 (94.4%)	
Has your doctor/family physician recommended you for an annual influenza vaccination?					0.038*			<0.001*
Yes	30 (10.1%)	5 (6.5%)	12 (9.0%)	13 (14.8%)		21 (17.5%)	9 (5.1%)	
No	268 (89.9%)	72 (93.5%)	121 (91.0%)	75 (85.2%)		99 (82.5%)	169 (94.9%)	
Main Source of Information on Influenza					0.002*			0.005*
Social media	94 (31.5%)	16 (20.8%)	42 (31.5%)	36 (40.9%)		49 (40.8%)	45 (25.3%)	
Printed media	7 (2.3%)	1 (1.3%)	5 (3.8%)	1 (2.3%)		2 (1.7%)	6 (3.4%)	
TV	70 (23.5%)	18 (23.4%)	40 (27.7%)	12 (27.3%)		31 (25.8%)	48 (27.0%)	
Friend	5 (1.7%)	0 (0.0%)	3 (2.3%)	2 (2.3%)		0 (0.0%)	5 (2.8%)	
Family	24 (8.1%)	2 (2.6%)	12 (9.0%)	10 (11.4%)		14 (11.7%)	10 (5.6%)	
Internet	23 (7.7%)	10 (13.0%)	10 (7.5%)	3 (3.4%)		6 (5.0%)	17 (9.6%)	
Radio	29 (9.7%)	7 (9.1%)	6 (4.5%)	16 (6.8%)		7 (5.8%)	12 (6.7%)	
Not applicable/refuse to answer	46 (15.4%)	23 (29.9%)	15 (11.3%)	8 (9.1%)		11 (9.2%)	35 (19.7%)	

**Figure 1:**
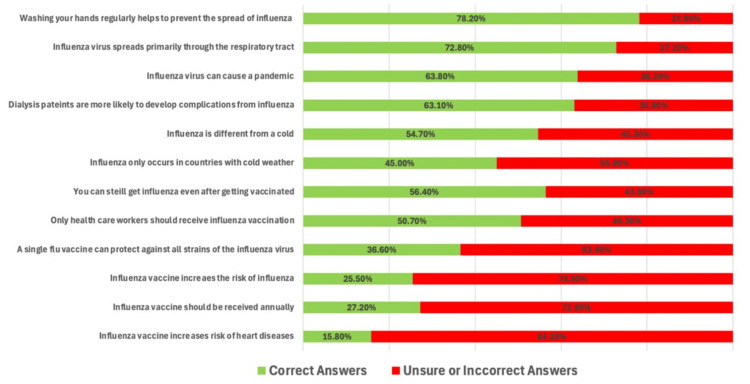
Bar chart showing correct or incorrect/unsure answers on questions on influenza and its vaccine

Our respondents were very aware that handwashing helps prevent the spread of influenza (78.2%), that influenza mainly spreads through the respiratory tract (72.8%), and that patients receiving hemodialysis have a higher risk of developing complications from an influenza infection (63.1%). Only 15.8% answered that influenza vaccines do not result in heart diseases, 25.5% that influenza vaccines do not weaken the immune system, and 27.2% that influenza vaccination should be administered annually in high-risk patients (Figure 1).

Only 40.6% of the respondents intended to receive influenza vaccination in the near future, with significant associations with ethnicity (χ2 = 11.226, df = 3, p=0.011), education level (χ2 = 13.798, df = 3, p=0.003), and income level (χ2 = 9.248, df = 3, p=0.01). There is also a significant association between knowledge level and the intention to receive an influenza vaccination (χ2 = 26.385, df = 2, p<0.01). Significant associations were detected between intention to vaccinate and all of the basic information, i.e., awareness of influenza (χ2 = 19.029, df = 2, p=0.005), history of vaccination (χ2 = 12.553, df = 2, p=0.002), previous advice from their doctor/family physician (χ2 = 12.259, df = 1, p<0.001), and their primary sources of information (χ2 = 20.828, df = 8, p=0.005) (Table 3).

**Table 3:** Chi-square associations of key predictors with intention to receive influenza vaccine

Variables	χ²	df	p-value
Ethnicity	11.226	3	0.011
Education level	13.798	3	0.003
Income level	9.248	3	0.010
Knowledge level	26.385	2	<0.001
Awareness of influenza	19.029	2	0.005
History of vaccination	12.553	2	0.002
Primary source of information	20.828	8	0.005
Previous advice from a doctor/family physician	12.259	1	<0.001

After adjusting for age, education level, religion, occupation, income group, awareness of influenza, and history of influenza vaccination, multivariate logistic regression showed that the best predictor for influenza vaccination is knowledge level, where moderate and reasonable knowledge levels are respectively 2.97 and 6.58 times more likely (95% CI 1.48-5.95, p≤0.002; 95% CI 3.15-13.89, p<0.001) to get vaccinated compared with those with poor knowledge levels. Others include recommendation by a family physician or own doctor (aOR=3.64, 95% CI 1.497-8.836, p=0.004), Chinese ethnicity (aOR=2.44, 95% CI 1.273-4.689, p=0.007), and female gender (aOR=1.72, 95% CI 1.018-2.915, p=0.043) (model fit was χ2 = 53.639, df = 7, p<0.001; Nagelkerke R^2^ = 0.215) (Table 4).

**Table 4:** Multivariate logistic regression analysis of independent variables * Statistically significant at p<0.05 OR: odds ratio, aOR: adjusted odds ratio, CI: confidence interval

	OR	95% CI	p-value	aOR	95% CI	p-value
Age, year	1.02	1.002-1.038	0.028			
Gender						
Male	Reference			Reference		
Female	1.434	0.898-2.294	0.132	1.721	1.018-2.915	0.043*
Ethnicity						
Malay	Reference			Reference		
Chinese	2.084	1.142-3.803	0.017	2.443	1.273-4.689	0.007*
Indian	2.648	1.082-6.481	0.033	2.423	0.940-6.251	0.06
Others	0.281	0.029-2747	0.275	3.175	0.216-47.619	0.4
Religion						
Islam	Reference					
Buddhist	2.143	1.091-4.208	0.027			
Hindu	2.23	0.948-5.246	0.066			
Christian	2.058	0.229-8.850	0.333			
Others	1.351	0.314-5.808	1.351			
Education						
No formal education	Reference					
Primary	5.882	0.693-50.000	0.104			
Secondary	6.579	0.828-52.632	0.075			
Tertiary	14.296	1.748-111.111	0.013			
Occupation						
Unemployed	Reference					
Self-employed	2.283	0.974-5.348	0.058			
Employee	3.663	0.816-3.663	0.153			
Retired	2.02	0.693-2.020	0.539			
Student	1.644	0.061-6.024	0.671			
Income						
B40	Reference					
M40	1.905	1.088-3.333	0.024			
T20	5.376	1.059-27.027	0.042			
Marital						
Single	Reference					
Married	1.166	0.632-2.152	0.623			
Divorced	1.983	0.550-7.147	0.295			
Widowed	1.388	0.497-3.875	0.531			
Center						
Private	Reference					
Government	1.05	0.658-1.678	0.837			
Have you heard of influenza?						
No	Reference					
Yes	3.068	1.361-6.918	0.007			
Has your doctor recommended you for an annual influenza vaccination?						
No	Reference			Reference		
Yes	3.983	1.756-9.038	<0.001	3.637	1.497-8.836	0.004*
Knowledge level						
Poor	Reference			Reference		
Moderate	2.653	1.368-5.155	0.004	2.967	1.479-5.952	<0.002*
Good	6.25	3.086-12.658	<0.001	6.579	3.145-13.889	<0.001*
Cox and Snell R^2 ^=0.159, Nagelkerke R^2 ^=0.215						

## Discussion

Main findings and international comparisons

This study reveals critically low influenza vaccination rates among patients receiving hemodialysis in Malaysia, with only 10.8% having ever received vaccination despite 90% awareness of influenza and its vaccine. Even more concerning, merely 3.4% receive annual vaccination, far below the WHO target of 75% for high-risk groups [19]. While 74.2% of participants demonstrated moderate to good knowledge levels about influenza, significant misconceptions persist regarding the safety and necessity of the vaccine. Only 40.3% expressed intention to vaccinate in the future, highlighting a substantial gap between knowledge and behavioral intention.

Our multivariate analysis identified three key predictors of vaccination intention: knowledge level (with good knowledge increasing the odds 6.58-fold), healthcare provider recommendation (a 3.64-fold increase), and female gender (a 1.8-fold increase). These findings underscore the complex interplay between knowledge, healthcare system factors, and individual characteristics in vaccination decision-making among this vulnerable population.

Our vaccination rate of 10.8% aligns with that of other low- and middle-income countries but falls dramatically short of the rates reported in high-income nations. European countries achieve an overall rate of 43.7%, with Portugal (60.8%), Spain (52.7%), and Italy (52.7%) demonstrating the impact of robust healthcare systems and vaccination policies [20-21]. Even among high-risk populations, previous Malaysian studies have reported vaccination rates of only 5-7%, suggesting systemic barriers rather than population-specific issues. The low vaccination rates can be attributed to the disparity in health policy implementation across different countries. Unlike in other countries, influenza vaccination in Malaysia for high-risk individuals was not mandatory until 2025 [13,22-24]. The implementation of mandatory vaccination policies significantly increases vaccination coverage in vulnerable populations, including those with chronic conditions or end-stage renal disease (ESRD) [22-25].

International literature consistently identifies healthcare provider recommendations as the strongest predictor of vaccination uptake, with rates ranging from 26.0% to 82.8% globally [23-24]. Our finding that only 10% of participants received provider recommendations represents a critical gap compared to high-income countries, where proactive provider engagement is standard practice. This disparity contributes significantly to the low vaccination rates observed in our population.

Understanding the knowledge-behavior gap

The moderate knowledge levels observed in our study (74.2% with moderate to good knowledge) contrast with those in studies from high-income countries, where knowledge levels are typically higher among patients with chronic diseases [20]. However, our findings align with studies from similar healthcare contexts, where awareness does not necessarily translate into vaccination uptake. The substantial disconnect between knowledge (74.2% moderate to good) and vaccination intention (40.3%) represents a critical finding that challenges the traditional assumption that knowledge directly translates into health behavior. This knowledge-behavior gap reflects the complex, multi-factorial nature of vaccination decision-making that extends beyond simple information processing.

The Health Belief Model provides a theoretical framework for understanding this gap, suggesting that health behaviors are influenced not only by knowledge but also by perceived susceptibility, severity, benefits, barriers, and cues to action [26]. Although our research did not further explore our participants' behavioral framework, our findings still align with this model, where, despite having adequate knowledge about influenza risks, participants may perceive themselves as having low personal susceptibility or facing high barriers to vaccination. The theory of planned behavior further explains this gap by highlighting how subjective norms (social expectations) and perceived behavioral control significantly influence intention formation beyond knowledge alone [27,28].

The persistence of vaccine misconceptions, despite overall high knowledge levels, suggests that emotional and cognitive biases play significant roles in decision-making. Risk perception theory suggests that individuals frequently rely on emotional, intuitive responses rather than analytical processing when assessing health threats [29,30]. Our finding that 70-85% of participants held misconceptions about vaccine safety demonstrates how fear-based information may override factual knowledge. This emotional dimension of decision-making is particularly relevant for vulnerable populations who may already experience heightened anxiety about their health status.

The knowledge-behavior gap may also reflect structural barriers that prevent intention from translating into action. Even when individuals intend to vaccinate, factors such as navigating the healthcare system, scheduling appointments, arranging transportation, and coordinating with dialysis schedules may prevent follow-through [31,32]. The low rate of provider recommendations (10%) suggests that the healthcare system itself may inadvertently create barriers by not facilitating vaccination opportunities during routine care.

Cultural and demographic factors

The finding that females were 1.8 times more likely to express vaccination intention reflects well-documented cultural patterns in Malaysian health-seeking behavior. In Malaysian society, women traditionally assume greater responsibility for family health management and are more likely to engage in preventive health behaviors [33]. This cultural role extends to personal health decisions, where women may feel greater social permission or expectation to seek medical interventions [34].

The association between Chinese ethnicity and higher vaccination intention (OR = 2.44) likely reflects several cultural factors specific to Malaysia's diverse population. Chinese Malaysian communities may have different cultural attitudes toward preventive medicine, influenced by both traditional Chinese medicine concepts of health maintenance and historical experiences with healthcare systems [35]. Additionally, Chinese Malaysian families may have stronger social networks that facilitate the sharing of health information and normalize preventive behaviors [36]. It's essential to note that these associations may also reflect socioeconomic factors, as ethnic groups in Malaysia have had varying historical access to education and healthcare resources.

The strong association between education level and vaccination intention must be understood within Malaysia's multicultural educational landscape. Higher education levels may reflect not only greater health literacy but also increased exposure to Western medical paradigms and evidence-based thinking [37,38]. However, this relationship may be complicated by cultural beliefs about health and illness that persist across educational levels. While not significant in our final model, the variation in vaccination intention across religious groups may reflect different theological perspectives on medical intervention and concepts of fate versus personal agency in health outcomes. Islamic teachings, for example, emphasize both trusting in Allah (*tawakkul*) and taking practical precautions (*asbab*), which may influence how Muslim patients approach vaccination decisions [39]. Understanding these cultural nuances is crucial for developing culturally sensitive vaccination programs.

Barriers to vaccination uptake

Three significant barriers emerge from our analysis: knowledge gaps, failures in the healthcare system, and the reliability of information sources. Despite overall moderate knowledge levels, critical misconceptions persist, particularly regarding vaccine safety. Many participants incorrectly believed vaccines could cause heart disease, weaken immune systems, or trigger influenza infection itself. These misconceptions, identified in 70-85% of responses to safety questions, represent significant barriers to vaccination acceptance. Concerns about immune system suppression are common among populations with existing health vulnerabilities, such as the immunocompromised or the elderly, despite consistent evidence supporting the safety and protective benefits of the influenza vaccine [40,41].

Healthcare system failures are evident in the low rate of provider recommendations (10%) and heavy reliance on social media for health information (31.5%). The absence of proactive healthcare provider engagement represents a missed opportunity, as provider recommendation emerged as one of the strongest predictors of vaccination intention in our multivariate model. This finding is particularly concerning, given that patients receiving hemodialysis have regular and frequent contact with healthcare providers. Worldwide studies reported that the recommendation rates of healthcare professionals recommending influenza vaccines vary between 26.0% and 82.8%, with high-income countries reporting a higher rate [42]. To date, physician recommendations remain one of the strongest predictors of vaccination uptake, as vaccination rates significantly increase when healthcare providers actively recommend them to patients [43-46].

The prevalence of social media as a source of information raises concerns about the quality and accuracy of the information it provides. Unlike traditional healthcare communication channels, social media content often lacks scientific credibility and may perpetuate misconceptions about vaccines [47]. This shift in information-seeking behavior requires healthcare systems to adapt their communication strategies to meet patients where they are accessing information.

Clinical and public health implications

Our findings have immediate implications for clinical practice and public health policy. The 6.6-fold increase in vaccination intention among participants with sound knowledge levels demonstrates the potential impact of targeted education interventions. Healthcare providers caring for patients receiving dialysis should prioritize discussions about influenza vaccination during routine care, as provider recommendations increased vaccination intention by 3.6 times. The gender disparity in vaccination intention (females 1.8 times more likely) suggests the need for tailored approaches for male patients, who may require different messaging or intervention strategies. This finding is particularly relevant given that chronic kidney disease disproportionately affects males in many populations [48,49].

From a public health perspective, the low vaccination rates in this high-risk population represent a significant missed opportunity for preventing influenza-related morbidity and mortality. Patients receiving hemodialysis face up to 10-fold higher mortality rates from influenza compared to the general population, making vaccination a critical protective intervention [1,2,5].

The regular healthcare contact inherent in hemodialysis care provides an ideal opportunity for systematic vaccination programs. As Malaysia has recently expanded its free influenza vaccination programs, several measures could be undertaken to increase vaccination rates, especially among patients receiving hemodialysis [50]. Vaccination protocols should be established in all dialysis centers, with regular discussions about influenza vaccination made mandatory during routine hemodialysis care by healthcare providers. These healthcare providers should also receive additional training and be empowered to provide effective vaccination counseling. Vaccination rates should be included as part of dialysis quality indicators. On the other hand, there should be development of culturally appropriate, multilingual educational materials addressing specific misconceptions on influenza vaccines, as identified in our study. Social media platforms should be used as a significant platform to deliver evidence-based health education, with successfully vaccinated patients serving as advocates or peer educators.

Study limitations

Several limitations should be considered while interpreting our findings. The cross-sectional design prevents causal inference, limiting our ability to determine whether improved knowledge directly leads to increased vaccination rates. Convenience sampling from five centers in Klang Valley may limit generalizability to other regions of Malaysia or different healthcare systems, particularly rural areas where access to healthcare and information may differ significantly. Nonetheless, in our study, we were able to sample the total population of ESRF patients across all five centers, allowing for a more complete and accurate representation of our target population.

Self-reported vaccination history introduces potential recall bias, as participants may inaccurately remember vaccination dates or details. The questionnaire format, although validated, may not fully capture the complexity of vaccination decision-making, including emotional, cultural, and familial influences. Despite controlling for multiple demographic and clinical variables, unmeasured confounders likely influence the observed associations. Factors such as health anxiety, previous adverse medical experiences, family influences, cultural beliefs, and personality traits may significantly impact vaccination decisions but were not captured in our quantitative survey. The modest explanatory power of our model (Nagelkerke R² = 0.215) suggests that substantial variation in vaccination intention remains unexplained by measured variables.

The study's focus on individual-level factors may underestimate the importance of structural barriers such as healthcare system capacity, vaccine availability, or financial constraints. Our study measured vaccination intention rather than actual vaccination behavior, introducing an additional layer of uncertainty. The intention-behavior gap in health psychology suggests that even strong intentions may not translate to actual vaccination due to practical barriers, changing circumstances, or competing priorities. This limitation is particularly relevant for patients receiving hemodialysis, who face multiple health challenges and may prioritize immediate concerns over preventive measures. Finally, the study was conducted before Malaysia expanded its free vaccination programs, so the findings may not accurately reflect current vaccination rates or intentions under the new policy environment.

Future research directions

Further research should focus on evaluating the effectiveness of various communication channels, particularly social media-based health education, as a public health communication strategy. Studies examining the impact of policy changes, such as Malaysia's expanded free vaccination program, would provide valuable insights into the effectiveness of structural interventions. Qualitative research exploring the cultural, family, and personal factors influencing vaccination decisions could provide a deeper understanding of barriers not captured in quantitative surveys.

Longitudinal studies tracking vaccination behavior over time help elucidate the relationship between knowledge, intention, and actual vaccination uptake. Furthermore, intervention studies testing culturally tailored education programs and healthcare provider training initiatives would provide evidence for effective strategies to improve vaccination rates in this vulnerable population.

## Conclusions

Despite its preliminary nature and limitations, our study exposes a life-threatening disparity between knowledge and vaccination uptake among hemodialysis patients in Malaysia, revealing a public health crisis with far-reaching implications for vulnerable populations. Despite possessing moderate knowledge levels and demonstrating high awareness of influenza's devastating potential, vaccination rates remain alarmingly inadequate at merely 10.8%, a figure that represents not just a statistic, but thousands of preventable deaths in a population already facing 10-fold higher mortality rates from influenza.

This catastrophically low vaccination rate stems from systemic failures, including persistent and dangerous misconceptions about vaccine safety, catastrophic gaps in healthcare provider engagement, and over-reliance on potentially misleading information sources. The human cost of inaction is unconscionable. Each unvaccinated hemodialysis patient represents a preventable hospitalization, a family devastated by loss, and healthcare resources strained by entirely avoidable complications.

As Malaysia expands its vaccination programs, this study provides an urgent blueprint for transformation. Success will require coordinated mobilization of public health agencies, healthcare providers, and communities to ensure that life-saving vaccines reach those teetering on the edge of survival. The stakes could not be higher: Immediate implementation of systematic vaccination programs could prevent catastrophic morbidity and mortality while dramatically improving the quality of life for thousands of patients with ESRD.

## Appendices

Part 1: screening criteria

1. Have you undergone dialysis for at least 3 months?

·         Yes - To proceed with questionnaire

·         No - Thank you for your participation

2. Have you been acutely ill in the past 2 weeks?

·         Yes - Thank you for your participation

·         No - To proceed with questionnaire

Part 2: sociodemographic data

Gender

·         Male

·         Female

Age __________

Ethnicity

·         Malay

·         Chinese

·         Indian

·         Others: __________

Religion

·         Islam

·         Buddhist

·         Hindu

·         Christian

·         Others: __________

Education level

·         No formal education

·         Primary

·         Secondary

·         Tertiary

Occupation

·         Unemployed

·         Self-employed

·         Employee

·         Retired

·         Student

Income level (per annum)

·         B40

·         M40

·         T20

Marital status

·         Single

·         Married

·         Divorced

·         Widowed

Basic information on influenza of the participants

Have you heard of influenza?

·         Yes

·         No

What is your main source of information on influenza? (Choose 1 only)

·         Social media

·         Printed media

·         TV

·         Radio

·         Internet

·         Friends

·         Family

·         Others

Do you receive influenza vaccinations annually?

·         Yes

·         Yes, but not annually

·         (Specify when was the last year you were vaccinated: __________)

·         Never

Are you willing to get vaccinated again in the near future?

·         Yes

·         No

Has your doctor/family physician(s) recommended you for annual influenza vaccination?

·         Yes

·         No

Part 3: clinical parameters

Duration of dialysis (years): __________

Cause for ESRF

·         Diabetes

·         Hypertension

·         Glomerular disease

·         Unknown

·         Others

Comorbidities

·         Hypertension

·         Diabetes

·         Heart diseases

·         Chronic lung diseases

·         Cancer

·         No

·         Others: (please specify) __________

Part 4: knowledge

1. Influenza virus spreads primarily through the respiratory tract (nose and mouth).

·         True

·         False

·         Unsure

2. Dialysis patients are more likely to develop complications from influenza infection.

·         True

·         False

·         Unsure

3. Influenza virus can cause a flu pandemic.

·         True

·         False

·         Unsure

4. Flu only occurs in countries with cold weather.

·         True

·         False

·         Unsure

5. Influenza is different from a cold.

·         True

·         False

·         Unsure

6. Washing your hands regularly helps to prevent the spread of influenza.

·         True

·         False

·         Unsure

7. Influenza vaccine SHOULD be received annually.

·         True

·         False

·         Unsure

8. Influenza vaccine can increase the risk of heart diseases.

·         True

·         False

·         Unsure

9. Only health care workers should receive influenza vaccination.

·         True

·         False

·         Unsure

10. You can still get influenza even after getting influenza vaccination.

·         True

·         False

·         Unsure

11. Influenza vaccine will weaken the immune system and increase the risk of getting influenza.

·         True

·         False

·         Unsure

12. A single flu vaccine can protect against all strains of the influenza virus.

·         True

·         False

·         Unsure
